# Strain-dependent Differences of Locomotor Activity and Hippocampus-dependent Learning and Memory in Mice

**DOI:** 10.5487/TR.2008.24.3.183

**Published:** 2008-09-01

**Authors:** Joong-Sun Kim, Miyoung Yang, Yeonghoon Son, Sung-Ho Kim, Jong-Choon Kim, Seungjoon Kim, Yongduk Lee, Taekyun Shin, Changjong Moon

**Affiliations:** 14 Department of Veterinary Anatomy, College of Veterinary Medicine, Chonnam National University, 300 Yongbong-dong, Buk-gu, Gwangju, 500-757 Korea; 24 Department of Veterinary Toxicology, College of Veterinary Medicine and Veterinary Medical Research Center, Chonnam National University, Gwangju, 500-757 Korea; 34 Department of Veterinary Obstetrics, College of Veterinary Medicine, Kyungpook National University, Daegu, 702-701 Korea; 44 Department of Veterinary Anatomy, College of Veterinary Medicine, Cheju National University, Jeju, 690-756 Korea

**Keywords:** Behavioral difference, Mouse strain, Locomotor activity, Passive avoidance, Object recognition memory

## Abstract

The behavioral phenotypes of out-bred ICR mice were compared with those of in-bred C57BL/6 and BALB/c mice. In particular, this study examined the locomotor activity and two forms of hippocampus-dependent learning paradigms, passive avoidance and object recognition memory. The basal open-field activity of the ICR strain was greater than that of the C57BL/6 and BALB/c strains. In the passive avoidance task, all the mice showed a significant increase in the cross-over latency when tested 24 hours after training. The strength of memory retention in the ICR mice was relatively weak and measurable, as indicated by the shorter cross-over latency than the C57BL/6 and BALB/c mice. In the object recognition memory test, all strains had a significant preference for the novel object during testing. The index for the preference of a novel object was lower for the ICR and BALB/c mice. Nevertheless, the variance and the standard deviation in these strains were comparable. Overall, these results confirm the strain differences on locomotor activity and hippocampus-dependent learning and memory in mice.

## INTRODUCTION

Mouse strains have characteristic differences according to their unique genetic make up. These differences can manifest in a wide variety of neurobiological traits including behavior (Crawley *et al*., 1997). Many studies have identified behavioral differences between mouse strains (Nguyen *et al*., 2000; Adams *et al*., 2002; Bouwknecht and Paylor, 2002; Brown and Wong, 2007). The requirement for multiple experimental control groups in genetic comparison studies highlights the need for reliable, instrument-directed, and learning assessment paradigms.

New developments in the control of genetic material and the mapping of widely used in-bred mouse strains have revived interest in mouse models of the behavioral function (Bucan and Abel, 2002). Therefore, behavioral data on in-bred mouse strains, including C57BL/6 and BALB/c, continues to be of interest because these strains are popular backgrounds for knockouts and mutants. On the other hand, the ICR out-bred strain has received less attention because it is generally accepted that a variable genetic background can complicate the measurements of certain behavioral traits. However, in reality, all clinical related studies need to consider the complication of genetic variability. Therefore, there is an emerging demand for establishing out-bred rodent models. Furthermore, it may be advantageous to use out-bred strains because they are robust, easy to breed, and resistant to diseases (Adams *et al*., 2002).

This study examined whether or not the out-bred ICR strain is suitable for studying certain aspects of the hippocampus functions. The ICR mice were examined using open-field activity and two learning paradigms, including passive avoidance and object recognition memory. Both passive avoidance and object recognition memory tasks can detect changes in the function of the hippocampus (Reed and Squire, 1997; Myhrer, 1998; Wang *et al*., 2004; Cimadevilla *et al*., 2007; Kim *et al*., 2008).

C57BL/6 and BALB/c mice were included in the behavioral analysis because the behavioral phenotypes of these two strains are well documented (Royle *et al*., 1999; Tsai *et al*., 2006; Van Dam *et al*., 2006; Depino and Gross, 2007). A comparison between the ICR and these two in-bred strains will also help clarify the effects of the genetic background on the locomotor activity and hippocampus-dependent learning and memory.

## MATERIALS AND METHODS

**Animals and tissue preparation.** Seven week old out-bred ICR (n = 44), in-bred C57BL/6 (n = 44) and BALB/c (n = 49) male mice were obtained from the animal center at the Korea Research Institute of Bioscience and Biotechnology and were bred in our animal facility. The animals were housed in a room maintained at a temperature of 23 ± 2°C, a relative humidity of 50 ± 5%, artificial lighting from 08:00 to 20:00, and 13~18 air changes per hour. All animal experiments followed a protocol approved by the Committee for Animal Experimentation at the Chonnam National University (Approval No.: CNU IACUC-YB-2008-6). One week before the behavioral examinations, the mice were handled (2~3 min) twice daily.

**Open-field test.** The open-field analysis was used to measure the activity of the mice in a novel environment. The 8-week-old mice were placed individually in brightly lit arenas (40 cm × 40 cm, 250 lux), which were equipped with automated infrared photocells to measure the activity. Parameters, including the total moving distance (cm), resting time (sec), ambulatory movement time (sec), and ambulatory movement episodes, were determined over a 10 min period using the Activity Monitor (MED-associates Inc, VT, USA).

**Passive avoidance.** The passive avoidance paradigm was used to examine the hippocampus-dependent association memory (Wang *et al*., 2004; Kim *et al*., 2008), in which the animals learned to associate the aversive unconditioned stimulus (mild electric foot shock) with the conditioned stimulus (the dark chamber). During training, a mouse (8-week-old) was introduced to the lit half of the training chamber (Ugo Basile, Italy), and allowed 1 min to explore the area before the trap door was opened. The trap door was closed, and a mild foot-shock (0.5 mA for 1 sec) was delivered immediately after the mouse had entered the darkened half. The trained mouse remained in the dark chamber for 20 sec after the shock, and was then returned to their home cage. When tested 24 hours after training, the trained mouse was re-introduced to the lit chamber. The time spent in the lit half before entering the darkened half was scored as the cross-over latency, and used as an index for memory formation. Five hundred seconds was chosen as the cut-off value for the cross-over latency (Kim *et al*., 2008). The mice were removed manually from the lit chamber when the cut-off value was reached.

The sensitivity to the electric foot-shock was further tested in mice. The electric shock thresholds (mA) to elicit stereotypic responses (flinch, vocalization, and jump/vocalization) were measured.

**Object recognition memory test.** Another hippocampus-dependent learning paradigm was used (Wang *et al*., 2004; Kim *et al*., 2008). The mice (8-week-old) were first habituated in the training/testing chamber (41.6 cm L, 27.6 cm W, 17.8 cm H) for 24 hours. The objects for recognition to be discriminated-made of plastic-ware in three different shapes: cubes, pyramids and cylinders 3.5 cm high; they could not be displaced by mice. The chamber arena and objects were cleaned by 75% ethanol between trials to prevent a build-up of olfactory cues. During training, two objects with different shapes were presented to each mouse for 15 min. Twenty-four hours after training, another set of objects (one old object and one novel object) was presented to the trained mouse. The interaction of mouse with each object, including approaches and sniffing was scored. If the mouse had memory retention for the old object, it would show preference to the novel object during testing. The percentage of preference was defined as the "number of interactions for a specific object" divided by the "total number of interactions for both objects".

**Statistical analysis.** The data is reported as the mean ± SE. The results were analyzed using one-way analysis of variance (ANOVA) followed by a Student-Newman-Keuls post hoc test for multiple comparisons. In all cases, a $p$ value < 0.05 was considered significant.

## RESULTS

**Open-field locomotion.** The basic locomotor activities of the ICR (n = 7), C57BL/6 (n = 6) and BALB/c (n = 8) mice in a novel environment were examined by open-field analysis. Compared with the C57BL/6 and BALB/c mice, the ICR mice showed a longer moving

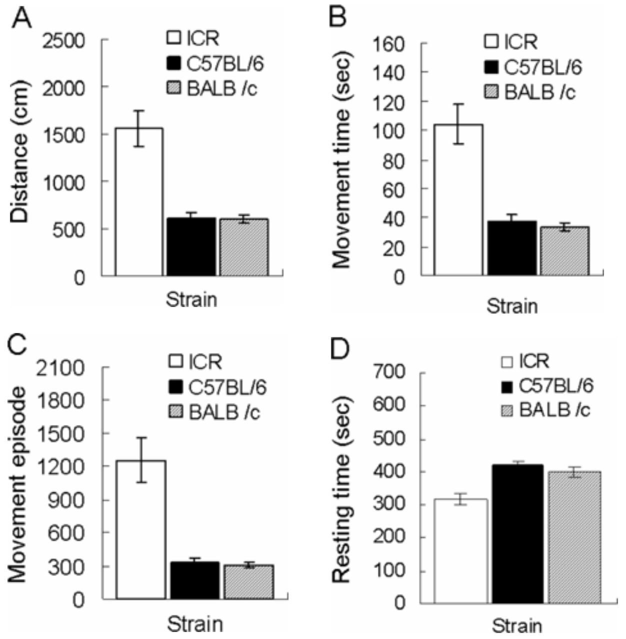

**Fig. 1.** Performance of the ICR, C57BL/6 and BALB/c in the open-field test. The data for the ICR (n = 7), C57BL/6 (n = 6) and BALB/c (n = 8) were obtained from an automated monitoring system. A. The ICR mice showed a longer movement distance (cm) than the C57BL/6 and BALB/c mice ($p < 0.001$ *vs.* both C57BL/6 and BALB/c). B. The ICR mice showed shorter resting time (sec) than the C57BL/6 and BALB/c mice ($p < 0.001$ *vs.* both C57BL/6 and BALB/c). C. The ICR mice showed a longer ambulatory movement time (sec) than the C57BL/6 and BALB/c mice ($p < 0.01$ *vs.* both C57BL/6 and BALB/c). D. The ICR mice showed a higher level of ambulatory movement episodes than the C57BL/6 and BALB/c ($p < 0.01$ *vs.* both C57BL/6 and BALB/c). The data is reported as the mean ± SE.

distance (Fig. 1A), a longer ambulatory movement time (Fig. 1B), and more frequent ambulatory movement episodes (Fig. 1C). Consistently, the ICR strain showed less resting time (Fig. 1D), which suggests that the basal locomotor activity of the ICR strain is higher than that of the C57BL/6 and BALB/c strains.

**Passive avoidance.** The ICR (n = 9), C57BL/6 (n = 8) and BALB/c (n = 9) mice were examined by passive avoidance. All mice subjected to this task stepped through the door into a dark compartment for a short period during the acquisition trial (33.3 ± 3.4, 61.8 ± 20.4 and 45.9 ± 4.71 sec for the ICR, C57BL/6 and BALB/c mice, respectively). There were no significant differences in cross-over latency during the acquisition trial between the three mouse strains (Fig. 2A).

The memory retention trials (testing) were carried out 24 hours after the acquisition trial (training). All the ICR (318.1 ± 38.4 sec), C57BL/6 (472.4 ± 27.6 sec) and BALB/c (479.8 ± 17.2 sec) mice showed a significant increase in cross-over latency when tested 24 hours after training (Fig. 2A). However, the ICR mice showed significantly shorter cross-over latency during the tests than the C57BL/6b and BALB/c (ICR *vs.* C57BL/6, $p < 0.01$; ICR *vs.* BALB/c, $p < 0.001$). In addition, the percentage of mice reaching the cut-off cross-over latency value was lower for the ICR mice (22.2%) than for the C57BL/6 (87.5%) and BALB/c (77.8%) mice (Fig. 2B). Because the ICR mice also showed lower cross-over latency during training, their lower cross-over latency during testing may not necessarily reflect their memory. The phenotype in the ICR mice may be partly due to

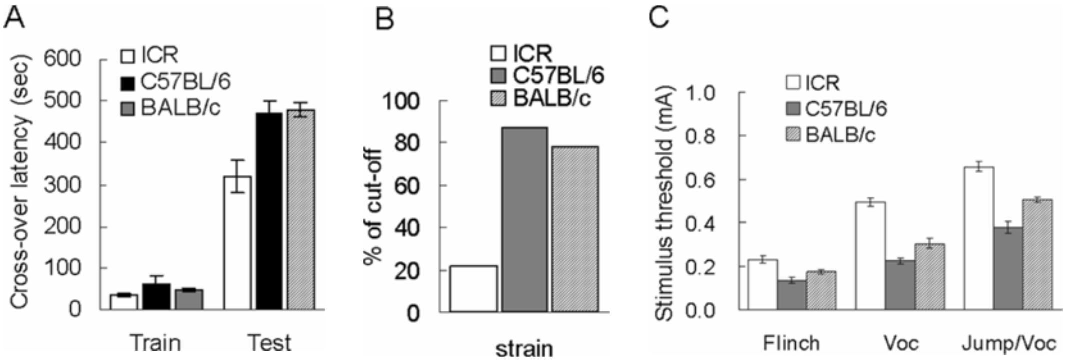

**Fig. 2.** Performance of the ICR, C57BL/6 and BALB/c mice in passive avoidance. A. The ICR, C57BL/6 and BALB/c mice showed a significant increase in cross-over latency during testing (24 hours after training). The ICR mice (n = 9) showed lower values for cross-over latency than the C57BL/6 (n = 8) and BALB/c mice (n = 9) when tested for passive avoidance 24 hours after training (ICR *vs.* C57BL/6, $p < 0.01$; ICR *vs.* BALB/c, $p < 0.001$). B. A lower percentage of ICR mice reached the cut-off value (500 sec) of the cross over latency during test than the C57BL/6 (87.5%) and BALB/c mice (77.8%). C. The electric shock thresholds (mA) to elicit stereotypic responses (flinch, vocalization, and jump/vocalization) were measured for the ICR (n = 8), C57BL/6 (n = 10) and BALB/c (n = 10) mice. The electric shock threshold of the ICR mice was significantly higher than those of the other strains, and the C57BL/6 mice had the lowest threshold for the shock. The data is reported as the mean ± SE (for A and C).

their higher level of locomotion.

The sensitivity to the electric foot-shock was further tested in ICR (n = 8), C57BL/6 (n = 10) and BALB/c (n = 10) mice, and there was a difference in the threshold current to elicit stereotypic responses including flinch, vocalization, and jump/vocalization between the three mouse strains: the C57BL/6 mice showed significantly higher sensitivity to electric shock than the other strains with the ICR mice showing lowest sensitivity (Fig. 2C). This result indicates that the anxiety and pain threshold was higher for the ICR mice than for the C57BL/6 and BALB/c mice.

**Object recognition memory.** ICR (n = 9), C57BL/6 (n = 7) and BALB/c (n = 9) mice was further examined using object recognition memory test. During training, two objects were presented to each mouse. During testing, one of these objects was replaced with a novel object. If the mouse remembered the old object, it would be expected to spend more time with the novel object, as indicated by a higher percentage of object preference.

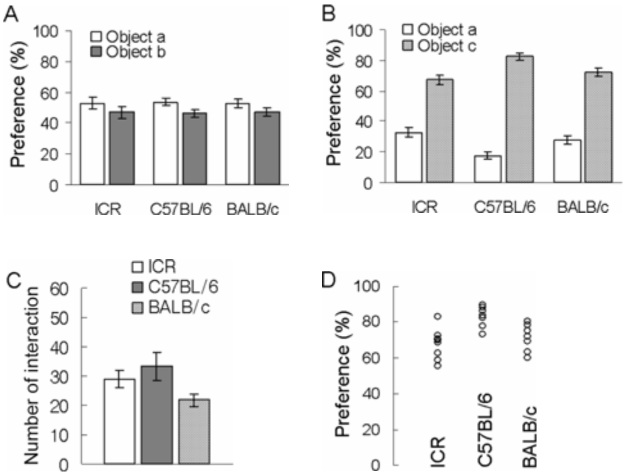

**Fig. 3.** Performance of the ICR, C57BL/6 and BALB/c mice in the object recognition memory test. The ICR (n = 9), C57BL/6 (n = 7), and BALB/c (n = 9) mice were examined. During training, two objects were presented to each mouse for 15 min. After 24 hours, one of the old objects was replaced with a novel object. If the mouse remembered the old object, it would spend more time with the novel object, as indicated by the higher percentage of object preference. A. The C57BL/6 mice showed a higher interaction with the two objects during training than the BALB/c mice ($p < 0.05$) but there was no significant difference in the interaction between the C57BL/6 and ICR mice. B. The ICR, C57BL/6 and BALB/c mice showed equal preference to the two objects during training. C. The ICR, C57BL/6 and BALB/c mice showed a significant preference to the novel object during testing (24 hours after training). However, the preference index was significantly higher for the C57BL/6 mice than the ICR and BALB/c mice (C57BL/6 *vs.* ICR, $p < 0.001$; C57BL/6 *vs.* BALB/c, $p < 0.01$). D. The individual distributions of the ICR, C57BL/6, and BALB/c mice show a similar variance. The data is reported as the mean ± SE.

During the 15 min training session, The ICR, C57BL/6 and BALB/c mice displayed equal preference to the two objects during training (Fig. 3A).

When tested 24 hours after training, all strains showed a high preference for the novel object. However, statistical analysis comparing the recognition indices between the strains obtained in the retention sessions indicated that the ICR mice (67.1 ± 3.0%) had a lower preference than the C57BL/6 (82.5 ± 2.5%) and BALB/c (72.1 ± 2.7%) mice (Fig. 3B).

In Figure 3C, the C57BL/6 mice showed higher interaction with the two objects during training, than the BALB/c ($p < 0.05$) mice. However, there was no significant difference in the interaction ($p = 0.407$) between the ICR and C57BL/6 mice. The weaker memory retention in the ICR mice may not be directly related to their interaction with the objects during training. The ICR mice exhibited a lower object interaction than the C57BL/6 mice but a higher object interaction than the BALB/c mice.

The distribution of the preference to a novel object for an individual mouse was compared because the variability in genetic background can cause more behavioral deviation in the out-bred ICR mice. However, it was found that the three strains showed a similar variance in the memory retention of the individual mice (Fig. 3D).

## DISCUSSION

This study demonstrates a difference in the locomotor activity and hippocampus-dependent memory retention between the out-bred ICR strain and the two widely used in-bred strains, C57BL/6 and BALB/c.

The open-field test is one of the oldest, most extensively used, and simplest measures for the emotional behavior of both mice and rats. An increasing illumination level or background noise in the open-field generally increases the stress level of these animals, resulting in decreased activity. Environmental conditions and the physical experiences, such as handling, stress and drug treatments, can affect the performance of an animal in the open-field activity (Crawley *et al.*, 1997). The open-field parameters all appear to reflect multiple underlying traits. The genes linked to the open-field performance might be involved in regulating the general locomotor activity, exploratory activity, olfaction and vision, as well as fear and anxiety (Crawley *et al.*, 1997). The albino strains, including ICR and BALB/c, took a longer time or more trials to learn the tasks, including Morris water maze and contextual fear conditioning, than the mouse strains with normal vision (C57BL/6) (Adams *et al.*, 2002; Brown and Wong, 2007). Nevertheless, in this study, the basal locomotor activity of the ICR mice was higher than those of the C57BL/6 mice. This suggests that compared with the C57BL/6 and BALB/c strains, the ICR strain has a higher level of general locomotor activity, exploratory activity and olfaction.

During passive avoidance training, the animals establish memory by associating the neutral conditioned stimulus (the dark chamber) with an aversive unconditioned stimulus (the foot-shock). In many cases, the floor of the apparatus is a grid, through which a mild electric foot-shock can be delivered. The animal receives an electric shock after entering a dark and presumably more comfortable environment. The passive avoidance task has been used in many learning and memory studies because strong memory retention can be achieved after a single training trial (Cimadevilla *et al.*, 2007). However, there are certain limitations for this paradigm: the passive avoidance test might be influenced by the anxiety and pain threshold (Yoneoka *et al.*, 1999). In this study, all three strains showed strong learning and memory retention in the passive avoidance task. However, the cross-over latency for the ICR mice was lower than the C57BL/6 and BALB/c strains. Furthermore, more C57BL/6 (87.5%) and BALB/c mice (77.8%) remained in the lit half for more than 500 sec (the cut-off value). In contrast, only 22.2% of the ICR mice reached the cut-off time. This difference might be due to the higher exploratory and locomotion activity (see Fig. 1), as well as to the low sensitivity to and fear of an electric foot-shock (Adams *et al.*, 2002). Therefore, ICR mice might be one of the choices for measuring the mild cognitive impairments induced by low-level effectors, such as low-dose irradiation or drugs with subtle side-effects on the neuronal function (Wozniak *et al.*, 1996; Zhao *et al.*, 2007; Kim *et al.*, 2008). In addition, a drug- or treatment-induced functional abnormality in the brain may be easier to detect because their memory retention for the passive avoidance paradigm is relatively weak and measurable.

The object recognition memory task is based on the spontaneous tendency of rodents to explore novel objects over familiar objects. Memory formation depends on both the hippocampus and the nigrostriatal dopaminergic pathway (Rampon *et al.*, 2000; Mumby, 2001; Schröder *et al.*, 2003), and does not require spatial information or awards/punishments. The distinction between true learning differences versus sensory impairments that lead to poor performance has been evaluated in different mouse strains (Crawley *et al.*, 1997). Brown and Wong (2007) reported consistent differences in visual detection according to the strain. These differences were predicted by the physiology of the visual system. In particular, mice with normal vision (such as C57BL/6 mice) performed very well in these tasks. In contrast, the albino strains, including BALB/c, took a longer time or more trials to learn the tasks than the mouse strains with normal vision. In this study, during the training session, the C57BL/6 mice showed higher level of interaction with the two training objects than the ICR and BALB/c mice. The C57BL/6 mice showed a higher preference to a novel object than the ICR and BALB/c mice during the test even though the ICR and BALB/c also showed a significant novel object preference. Further analysis/examination might be required to determine the visual function, as well as the neuronal structures in the hippocampus and the nigrostriatal dopaminergic pathway of the ICR mice. Nevertheless, the ICR mice showed a significant interaction with the objects during training and testing, indicating sufficient visual and hippocampal function. The three strains also showed similar variance in the memory retention of individual mice. Therefore, the ICR mice show stable learning and memory formation, and are considered suitable for learning and memory studies. However, more studies will be needed to establish the relationship between genetic strain, synaptic plasticity and memory formation.

In conclusion, this study confirmed that the differences in locomotor activity and hippocampus-dependent memory behavior are related to the genetic background. The out-bred ICR mice, which have gained popularity in drug and pre-clinical research (Wozniak *et al.*, 1996; Zhao *et al.*, 2007), displayed significant and measurable hippocampal-dependent memory formation.

## ACKNOWLEDGEMENTS

This study was supported by the Ministry of Education, Science and Technology of Korea through the Nuclear R&D Program and the Regional Research Centers Program (Bio-housing Research Institute).
